# Training of Pugilists

**Published:** 1860-08

**Authors:** 


☞ We take the following, on the regimen and physical training of pugilists, from an editorial in the *London Lancet:*

"The professors of the art accord, we believe, an enlightened respect to the precepts of medicine. We are, however, unable to describe their practice from personal experience. It is understood that the plan pursued is varied, according to the condition and requirements of the individual in training. But the general system is somewhat as follows: Diet and exercise are most scrupulously regulated. The athlete, who has, by indulgence or inaction, become too fat and short-winded, is thickly clad in woollen garments, and made to run long distances, frequently up hill, until he perspires freely. He is then carefully rubbed with coarse towels. He also takes frequent baths, so that the skin is thoroughly cleansed, and made to perform its emunctory and other functions with the greatest perfection. He is submitted to a variety of exercise, such as sparring, the use of dumb-bells, and other means calculated to increase muscular development, and expand the chest. His principal food consists of beefsteaks and mutton-chops. The steaks are well beaten to render the muscular fibre more digestible; dressed in a frying pan, diligently polished to exclude the chance of dirt, or other contamination; and cut into very thin morsels to facilitate mastication, and that minute subdivision which is conducive to perfect digestion. He is not restricted from beer, but is compelled to be moderate. Under a few weeks' subjection to this system, the puffy fellow, who could not run twenty yards without panting, nor receive a moderate blow without exhibiting bruises and extravasations, is disincumbered of all superfluous tissue, and brought into a condition capable of the greatest physical exertion and endurance. The regenerated athlete comes into the ring, exulting in muscular power and activity. Suppleness and force are revealed in every movement. You have before you the ideal of the human animal personified. Nor is the result entirely one of animal excellence. The physical qualities of man can hardly be wrought to a high pitch without also evoking some of the moral good that is in him. The training itself implies mental as well as physical discipline. For a long time the pugilist having his aim in view, possibly a bad aim, has exercised the most resolute self-denial. When he encounters his antagonist, that self-denial gives place to perfect self-control. We fear we must not divert the word chivalry from its ordinary acceptation to conflicts of this kind, but there is surely something akin to it in that unswerving and not ungenerous observance of the rules of "fair play," and that admirable command of temper under the most severe punishment, which are amongst the characteristics of the professional pugilist. Now when we remember that men and boys will quarrel and fight, it is impossible not to recognise the utility of some standard that shall serve to control and moderate the brutal passions of combatants. This we undoubtedly see. The laws of the ring exert their sway over the whole population, and unquestionably often prevent acts of cowardice and cruelty."

